# Correction: Peavey et al. Nuclear Receptor Atlases of Choroidal Tissues Reveal Candidate Receptors Associated with Age-Related Macular Degeneration. *Cells* 2022, *11*, 2386

**DOI:** 10.3390/cells11243948

**Published:** 2022-12-07

**Authors:** Jeremy Peavey, Vipul M. Parmar, Goldis Malek

**Affiliations:** 1 Duke Eye Center, Department of Ophthalmology, Duke University School of Medicine, Durham, NC 27710, USA; 2 Department of Pathology, Duke University School of Medicine, Durham, NC 27710, USA

In the original publication [1], there was a mistake in Figure 2 as published. The authors identified a typo in panel F of this figure. The nuclear receptor RORβ is listed as absent from primary CEC culture, whereas RXRβ is actually absent. The corrected Figure 2 appears below.

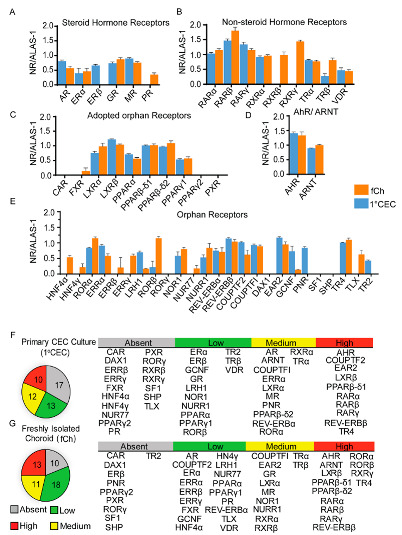



The authors state that the scientific conclusions are unaffected. This correction was approved by the Academic Editor. The original publication has also been updated.
