# Supplementary material for: Nuclear Receptor Atlases of Choroidal Tissues Reveal Candidate Receptors Associated with Age-Related Macular Degeneration
Source: Cells. 2022 Aug 2;11(15):2386. doi: 10.3390/cells11152386 (PMC9367936; doi:10.3390/cells11152386)
Supplement: Supplementary file 1 [file cells-11-02386-s001.zip › cells-1779907-supplementary.pdf]

Supplemental Figure S1: Heatmap of differentially regulated nuclear receptors in the choroid of aged laser induced CNV mice versus controls, for visualization of log2 (fold change).

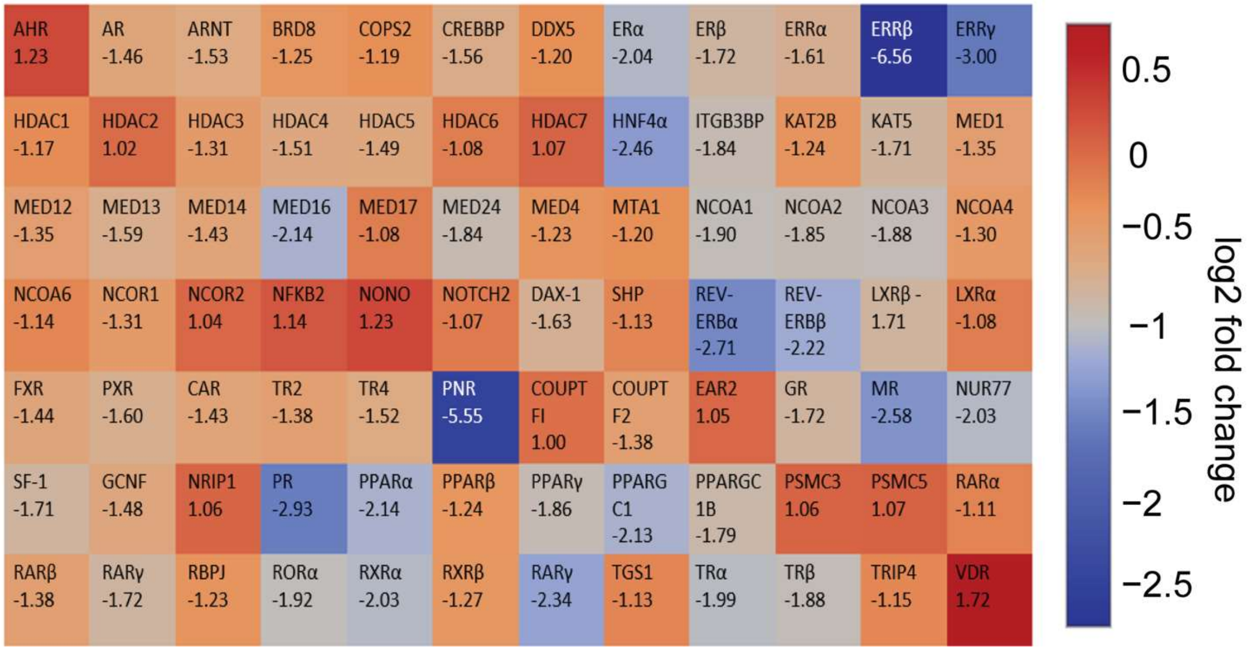

Supplemental Table S1. Demographics of human donor tissues from which choroidal endothelial cells were isolated to establish cell culture lines.

Donor	Age	Gender	Race/Ethnicity	Highest passage	Death to culture (hours)	Smoking status	Cause of death
1900-1602	47	Male	Hispanic or Latino	≤5	≤8	Never	Unknown
1900-1352	52	Male	Asian	≤5	≤8	Never	Unknown
1900-1737	57	Male	Caucasian	≤5	≤8	Quit; 2019	Esophageal Cancer
1900-0994	66	Male	African American	≤5	≤8	Never	Respiratory Failure
1900-1413	75	Female	Caucasian	≤5	≤8	Never	Organ Failure
1900-1609	79	Male	American Indian	≤5	≤8	Quit; 1972	Parkinson's disease, Sepsis
1900-1611	84	Male	Caucasian	≤5	≤8	Quit; 1970	Unknown
1900-1621	90+	Female	Caucasian	≤5	≤8	Quit	Cardiogenic Shock

Supplemental Table S2. Demographics of human donor tissues from which fresh choroid was isolated.

Donor	Age	Gender	Race/Ethnicity	Death to culture (hours)	Smoking status	Cause of death
1441-12-04	61	Female	Caucasian	≤8	Unknown	Uterine cancer
1427-12-03	69	Male	Caucasian	≤8	Yes	Pancreatic cancer
1384-12-04	74	Female	Caucasian	≤8	Unknown	Heart disease
12-1346	74	Male	Caucasian	≤8	No	Subdural Hematoma
13-0535	86	Male	Caucasian	≤8	Unknown	Pulmonary embolism
12-1521	90	Female	Caucasian	≤8	Unknown	Congestive Heart Failure

Supplement Table S3. Genotype analysis of the human donor samples from which cell culture lines were established.

Donor	CFH (rs1061170)	CFI (rs10033900)	C2 (rs9332739)	C3 (rs2230199)	CFB (rs4151667)	ARMS2 (rs10490924)	HTRA1 (rs2372598)	APOE (rs429358)	APOE (rs7412)	APOE isotype
**1900-1602**	TT; Homo	CC; WT	GG; WT	GG; WT	TT; WT	GG; WT	TC; Het	CT	CC	E2/E3
**1900-1609**	CT; Het	CT; Het	GC; Het	GG; WT	TA; Het	GG; WT	TT; WT	TT	CC	E3/E3
**1900-1611**	CT; Het	CC; WT	GG; WT	GG; WT	TT; WT	GG; WT	CC; Homo	TT	CC	E3/E3
**1900-1621**	TT; Homo	CT; Het	GG; WT	GG; WT	TT; WT	GG; WT	TT; WT	TT	CC	E3/E3
**1900-1737**	CT; Het	CC; WT	GG; WT	GC; Het	TT; WT	GG; WT	TC; Het	TT	CC	E3/E3
**1900-1413**	TT; Homo	TT; Homo	GG; WT	GG; WT	TT; WT	GG; WT	TC; Het	CT	CC	E2/E3
**1900-0994**	CT; Het	CT; Het	GG; WT	GG; WT	TT; WT	GT; Het	TC; Het	TT	CC	E3/E3
**1900-1352**	CT; Het	CT; Het	GC; Het	GG; WT	TA; Het	GT; Het	CC; Homo	TT	CC	E3/E3

Supplemental Table S4. Nuclear receptor gene symbol, unified nomenclature, and common name.

	Gene	Unified nomenclature	Common name
1	AhR	AHR	Aryl Hydrocarbon Receptor
2	ARNT	ARNT	Aryl Hydrocarbon Receptor Nuclear Translocator
Steroid hormone receptors			
3	AR	NR3C4	Androgen Receptor
4	ERα	NR3A1	Estrogen Receptor α or Estrogen Receptor 1
5	ERβ	NR3A2	Estrogen Receptor β or Estrogen Receptor 2
6	GR	NR3C1	Glucocorticoid Receptor
7	MR	NR3C2	Mineralocorticoid Receptor
8	PR	NR3C3	Progesterone Receptor
Non-steroid nuclear receptors			
9	RARα	NR1B1	Retinoic Acid Receptor Alpha
10	RARβ	NR1B2	Retinoic Acid Receptor Beta
11	RARɣ	NR1B3	Retinoic Acid Receptor Gamma
12	RXRα	NR2B1	Retinoid X Receptor Alpha
13	RXRβ	NR2B2	Retinoid X Receptor Beta
14	RXRɣ	NR2B3	Retinoid X Receptor Gamma
15	TRα	NR1A1	Thyroid Hormone Receptor Alpha
16	TRβ	NR1A2	Thyroid Hormone Receptor Beta
17	VDR	NR1I1	Vitamin D Receptor
Orphan nuclear receptors			
18	COUP-TF2	NR2F2	Chicken Ovalbumin Upstream Promoter-Transcription Factor I
19	COUP-TFI	NR2F1	Chicken Ovalbumin Upstream Promoter-Transcription Factor 2
20	DAX-1	NR0B1	DSS-AHC Critical Region On The X Chromosome Protein 1
21	EAR-2	NR2F6	V-ErbA-Related Protein 2
22	ERRα	NR3B1	Estrogen Related Receptor Alpha
23	ERRβ	NR3B2	Estrogen Related Receptor Beta
24	ERRɣ	NR3B3	Estrogen Related Receptor Gamma
25	GCNF	NR6A1	Germ Cell Nuclear Factor (Retinoid Receptor-Related Testis-Specific Receptor)
26	HNF4α	NR2A1	Germ Cell Nuclear Factor (Retinoid Receptor-Related Testis-Specific Receptor)
27	HNF4ɣ	NR2A2	Hepatocyte Nuclear Factor 4 Alpha
28	LRH-1	NR5A2	Hepatocyte Nuclear Factor 4 Gamma
29	NOR1	NR4A3	Liver Receptor Homolog-1
30	NUR77	NR4A1	Neuron-Derived Orphan Receptor 1
31	NURR1	NR4A2	Nerve Growth Factor IB Nuclear Receptor Variant 1
32	PNR	NR2E3	Human Homolog Of Nur Related Protein-1
33	Rev-Erbα	NR1D1	Photoreceptor-Specific Nuclear Receptor
34	Rev-Erbβ	NR1D2	V-ErbA-Related Protein 1
35	RORα	NR1F1	V-ErbA-Related Protein 1-Related
36	RORβ	NR1F2	Retinoid-Related Orphan Receptor Alpha

37	RORɣ	NR1F3	Retinoid-Related Orphan Receptor Beta
38	SF-1	NR5A1	Retinoid-Related Orphan Receptor Gamma
39	SHP	NR0B2	Steroidogenic Factor-1
40	TLX	NR2E1	Small Heterodimer Partner
41	TR2	NR2C1	Nuclear receptor TLX
42	TR4	NR2C2	Testicular Receptor 2
Adopted Orphan nuclear receptors			
43	CAR	NR1I3	Constitutive Active Receptor
44	FXR	NR1H4	Farnesoid X Receptor
45	LXRα	NR1H3	Liver X Nuclear Receptor Alpha
46	LXRβ	NR1H2	Liver X Nuclear Receptor Beta
47	PPARɣ1	NR1C3	Peroxisome Proliferator Activated Receptor Gamma isoform 1
48	PPARɣ2	NR1C3	Peroxisome Proliferator Activated Receptor Gamma isoform 2
49	PPARα	NR1C1	Peroxisome Proliferator Activated Receptor Alpha
50	PPARβ/δ1	NR1C2	Peroxisome Proliferator Activated Receptor Delta 1
51	PPARβ/δ2	NR1C2	Peroxisome Proliferator Activated Receptor Delta 2
52	PXR	NR1I2	Pregnane X Nuclear Receptor Variant 2

Supplemental Table S5. Gene primer sequences.

	Gene	Forward primer	Reverse Primer
1	ALAS1	CGCCGCTGCCCATTCTTAT	TCTGTTGGACCTTGGCCTTAG
2	β-Actin	CTTCCTTCCTGGGCATGGACTC	GGGTACATGGTTGGTGCCGC
3	AhR	TCCACCTCAGTTGGCTTTGTTTGC	TCGTGCACAGCTCTGCTTCAGTAT
4	ARNT	CTGCCAACCCCGAAATGACAT	GCCGCTTAATAGCCCTCTGG
5	AR	CTGCGCTGACCTTAAAGACAT	TGCCCCCTAAGTAATTGTCCTT
6	ERα	AGAGAAGTATTCAAGGACATAACGACTATAT	TCTTCCTCCTGTTTTTATCAATGG
7	ERβ	AAGTTGGCCGACAAGGAGTT	ACAGGCTGAGCTCCACAAAG
8	GR	CCATTGTCAAGAGGGAAGGAAAC	ATGATTTCAGCTAACATCTCGGG
9	MR	CCAAATCAGCCTTCAGTTCGT	TTGAGGCCATCCTTTGGAAT
10	PR	TGGGAGCTGTAAGGTCTTCTTTAA	ACGATGCAGTCATTTCTTCCA
11	RARα	CAGCACCAGCTTCCAGTTAGTG	CTGCTGCTCTGGGTCTCAATG
12	RARβ	CAGCTCCTGCCTTTGGAAA	CTTTTGTCGGTTCCTCAAGGT
13	RARɣ	GGAACAAGAAGAAGAAAGAGGTGAA	TTGGTGATGAGCTCTTCTAACTGA
14	RXRɣ	GAAGTTTCCCGCAGGCTATG	TGATGGGCTCATGGATGTAGA
15	RXRα	GGACTGCCTGATTGACAAGC	TTCAGCCCCATGTTTGCCTC
16	RXRβ	AGCCCCCAGATTAACTCAACA	GATTGCACATAGCCGTTTGC
17	TRα	CACGGAAGTGGCTCTGCTG	GCAGGTACGCCTCCTGACTC
18	TRβ	TGCGTGGGTGCCAAGT	CCTTTTTTCACTGACATCTCCTTCT
19	VDR	CCCCACCTACTCCGACTTCT	CTCCACCATCATTCACACGAA
20	COUP-TF2	AAGGCGCTGCACGTTGAC	CTTTCCACATGCTCTCTACATCAG
21	COUP-TFI	ACAGCTGCCTCAAAGCCATC	TCACGTACTCCTCCAGTGCG
22	DAX-1	CCAGGTCCAAGCCATCAAGT	GGCACGTCCGGGTTAAAGA
23	EAR-2	AGGGCTGCAAGAGCTTTTTC	TGGCAGTCACGGTTGGA
24	ERRα	GCGAGAGGAGTATGTTCTACTAAAGG	AGCCTCGGCATCTTCGAT
25	ERRβ	GAGGACTATCCAAGGGAACATTG	CATCCCCACTTTGAGGCATT
26	ERRɣ	GCTAACACTGTCGCAGTTTGAA	CGAACAGCTGGAATCAATGTG
27	GCNF	ATCGAGCGGCTCATCTACCT	ATATCTTGATTTAGGAAGTTAATTGCTTTC
28	HNF4α	TGCAGGCTCAAGAAATGCTT	TCATTCTGGACGGCTTCCTT
29	HNF4ɣ	TGGGTGCAAGGGTTTCTTC	CCGACTGAACCTGCAAGAATA
30	LRH-1	TCGACCACATTTACCGACAAG	CCACTAACTCCTGTGCATGACT
31	NOR1	CTGAGCATGTGCAACAATTCTAC	ACAGCTCCAAAAAGGCTGATTC
32	NUR77	CCCTTCGTGCGGTTGTCT	GGCTTGGATCACGGGCATCT
33	NURR1	GCCCAAAGCCGACCAA	GGACCTGTATGCTAATCGAAGGA
34	PNR	TGTGCCCCGTGGACAA	CGGCGTCCTGGTTCATC
35	Rev-Erbα	TGACCAAGTC ACCCTGCTTAAG	AAGCAAAGCGCACCATCA
36	Rev-Erbβ	TCTTGTCACAGTGAGGGTTCT	GCGAGATCACCATTCTTGGGA
37	RORα	GCAGCCATGAGCGATCTG	GCAGCCATGAGCGATCTG
38	RORβ	TATTACAACGTCGATTCCGGTCA	GGTACGGATGTGAGGTCATAGA
39	RORɣ	GCAGCGCTCCAACATCTTC	GCACACCGTTCCCACATCT

40	SF-1	TTCTGCCGCTTCCAGAAAT	TTGTACATCGGCCCAAACTT
41	SHP	CCTGCCTGAAAGGGACCAT	CTGCAGGTGCCCAATGTG
42	TLX	TGATGCTAACACTCTACTGGCTGTA	CAGCTTCTGGGAATCTGTGTTG
43	TR2	GCAGACCAACGGTGATGTTT	CCAGGATTCAATGCTTTTGC
44	TR4	GATGGGCATGAAAATGGAATC	GGTTTCTCCCGTTGCACAT
45	CAR	TTCATGGTACTGCAAGTCATCAA	TTGAGAAGGGAGATCTGGTCTTC
46	FXR	TGTCGACTAAGGAAATGCAAAGA	TGCTGCTTCACATTTTTTCTCA
47	LXRα	CCCTTCAGAACCCACAGAGATC	GCTCGTTCCCCAGCATTTT
48	LXRβ	GTATTTGAGTAGCGGCGGTGTG	CTCCTGTTGCCCTGGGTTCC
49	PPARɣ1	AGATCCAGTGGTTGCAGATTACA	GGAGATGCACTCGCTCCACTTT
50	PPARɣ2	TGACCCAGAAAGCGATTCCT	CAAAGTTGGTGGGCCAGAA
51	PPARα	ACGTGCTTCCTGCTTCATAGAT	CACCATCGCGACCAGATG
52	PPARβ/δ1	AGAAGAACCGCAACAAGTGC	CTCCCCTCGTTTGCAGTCAG
53	PPARβ/δ2	CCAACAGAGTAAGACAGATGCA	CTGAACGCAGATGGACCTCTA
54	PXR	GCAAAGAACTTACCACCAAACAG	CCCCACATACACGGCAGATTT

Supplemental Table S6. Confirmation of nuclear receptors categorized as absent based on CT values.

	Gene		
	**DAX**	**ROR-γ**	**SHP**
Primary CECs Passage 3 (8 donors)	34.17	38.23	36.33
Freshly Isolated (6 donors)	36.92	33.08	41.62
